# Experience of Offering HIV Rapid Testing to At-Risk Patients in Community Health Centers in Eight Chinese Cities

**DOI:** 10.1371/journal.pone.0086609

**Published:** 2014-01-28

**Authors:** Dapeng Zhang, Sining Meng, Peng Xu, Hongyan Lu, Minghua Zhuang, Guohui Wu, Yanling Liu, Xiaohong Pan, Hongjing Yan, Xi Chen, Lirui Fan, Chengmei Li, Xiaojing Fu, Jinlei Qi, Lei Han, Fuchang Ma, Fan Lv, Jiangping Sun

**Affiliations:** 1 National Center for AIDS/STD Control and Prevention, Chinese Center for Disease Control and Prevention, Beijing, China; 2 Beijing Centers for Diseases Control and Prevention, Beijing, China; 3 Shanghai Municipal Center for Disease Control and Prevention, Shanghai, China; 4 Chongqing Center for Disease Control and Prevention, Chongqing, China; 5 Harbin Center for Disease Control and Prevention, Harbin, China; 6 Zhejiang Provincial Center for Disease Control and Prevention, Hangzhou, China; 7 Jiangsu Provincial Center for Disease Control and Prevention, Nanjing, China; 8 Hunan Provincial Center for Disease Control and Prevention, Changsha, China; 9 Guangzhou Center for Disease Control and Prevention, Guangzhou, China; 10 Shandong Provincial Center for Disease Control and Prevention, Jinan, China; Fundacion Huesped, Argentina

## Abstract

**Objective:**

To explore the feasibility of offering HIV counseling and testing in community health centers (CHCs) and to provide evidence for the HIV/AIDS response in China.

**Methods:**

Forty-two CHCs were selected from the eight cities that participated in the study. Rapid testing was mainly provided to: clients seeking HIV testing and counseling (HTC); outpatients with high-risk behavior of contracting HIV; inpatients and outpatients of key departments. Aggregate administrative data were collected in CHCs and general hospitals and differences between the two categories were compared.

**Results:**

There were 23,609 patients who underwent HIV testing, accounting for 0.37% of all estimated clinic visits at the 42 sites (0.03%–4.35% by site). Overall, positive screening prevalence was 0.41% (95% confidence interval [CI] 0.33%–0.49%, range 0.00%–0.98%), which is higher than in general hospitals (0.17%). The identification efficiency was 0.22% (95% CI: 0.16%–0.27%) in pilot CHCs, 3.5 times higher than in general hospitals (0.06%) (Chi square test = 95.196, p<0.001). The percentage of those receiving confirmatory tests among those who screened positive was slightly lower in CHCs (73.7%) than in general hospitals (80.1%) (Chi-square test = 17.472, p<0.001). Composition of clients mobilized for testing was consistent with the usage of basic public health and medical services in CHCs. The rate of patients testing HIV positive was higher among patients from key CHC departments (0.68%) than among high-risk Voluntary Counseling and Testing (VCT) clients (0.56%), those participating in outreach activities (0.41%), pregnant women (0.05%), and surgical patients (0.00%).

**Conclusion:**

This project demonstrates that providing HIV testing services for patients who exhibit high risk behavior has a high HIV case detection rate and that CHCs have the capacity to integrate HTC into routine work. It provides concrete evidence supporting the involvement of CHCs in the expansion of HIV/AIDS testing and case finding.

## Introduction

In the last decade, China's HIV epidemic has gradually increased. In 2011, it was estimated there were 780,000 people living with HIV/AIDS (PLHA), over 50% of them unaware of their infection status [1]. In 2010, the Chinese State Council issued an action plan to reduce HIV incidence by 25% and reduce AIDS-related mortality by 30% by 2015. A key strategy to achieve these goals is to expand HIV testing and counseling (HTC) in order to increase the proportion of people who know their status [2].

The HIV/AIDS response in China is primarily organized by Centers for Disease Control and Prevention (CDCs) and hospitals across governmental levels. Most HTC is provided by Voluntary Counseling and Testing (VCT) clinics based in CDCs and general hospitals. Hospitals also provide compulsory HTC for inpatients, surgical patients, pregnant women, or people who donate blood or who need a blood transfusion [3]. In some provinces, provider-initiated HIV testing and counseling (PITC) is conducted in general hospitals for outpatients in key departments, such as the urology, gynecology, perianal, and sexually transmitted infection (STI) departments [4]. Finally, with the support of international organizations, some local CDCs are collaborating with community-based organizations (CBOs) to conduct HTC mobilization with key populations, especially men who have sex with men (MSM) [5]. However, this model is currently facing bottlenecks and challenges, including heavy workloads for CDC staff, inconvenient timing and location of testing sites, hard-to-reach target populations, fear of stigma and discrimination, and urgent scale-up needs of HIV testing due to lack of HTC access among people at risk for HIV infection [3], [6], [7].

In 2006, the United States Center for Disease Control and Prevention (USCDC) published updated recommendations for HTC in health settings [8]. One notable change was the move to use “opt-out” testing, where all patients in health settings, excluding those who explicitly decline, should be informed and tested for HIV, regardless of signs, symptoms, or risk factors [9]. After this change, the role of health clinics at the community level in expanding access to HTC has been explored internationally, showing that integrating HTC into routine health work of CHCs can increase access to HTC [9], [10], [11], [12], [13], [14].

Historically, CHCs in China have provided comprehensive front-line medical and public health services [15]. Their on-the-ground experience gives them inherent advantages for the HIV/AIDS response [16]. The scale of CHCs (more than 33,000 in 2010) provides the opportunity for significant impact. The extensive grassroots networks of community health service providers allow them to incorporate HIV/AIDS activities into routine services in a sustainable way. Finally, the medical training of CHC staff gives them the capacity to effectively implement HIV/AIDS activities. Since 2009, the Chinese government has been conducting medical health system reform. Equalization of basic public health services is a key component of the reform [16]. CHCs are taking the leading role in providing primary public health services, which mainly include disease control and prevention, health care services, health education, family planning, medical treatment services and community rehabilitation [17]. Integrating the AIDS response into CHC routine services would be a huge benefit for AIDS prevention work in China. However, it is challenging in terms of what services related to AIDS prevention CHCs can provide and how to integrate them into CHC routine services.

Although there has been increasing acceptance of general hospitals playing a greater role in HIV/AIDS case finding in China, concerns remain about providing HTC at CHCs. First, CHCs primarily serve women, children, elderly, the disabled, chronic disease sufferers, and impoverished individuals at the community level. No publications have been found in China regarding the HIV infection rate among attendees of CHCs. The infection rate might be low, potentially lowering the cost efficiency of HTC. Second, there is uncertainty regarding CHC capacity to mobilize higher-risk attendees and providing quality counseling and follow-up services [18]. Third, there is the potential that testing at CHCs will not be accepted, due to patient fears of exposure, stigma, and discrimination [3].

Given the lack of prior research on the feasibility of providing systemic HIV testing in CHCs in China, we conducted a pilot project integrating HTC with routine CHC health services. The project explored ways to engage community health service providers in the HIV/AIDS response and provided evidence for the HIV/AIDS response in China.

## Methods

### Study procedure

The pilot included four to five CHCs per city in eight of the 15 China-Gates Foundation HIV project cities (Beijing, Shanghai, Chongqing, Harbin, Nanjing, Hangzhou, Changsha, Guangzhou) for a total of 42 CHCs. It lasted from November 2011 to December 2012. Before pilot implementation, only eight of the 42 CHCs participated in HIV/AIDS work, focusing on health education and female sex worker (FSW) intervention. Five of these CHCs provided HTC to inpatients, surgical patients, and pregnant women.

Pilot CHCs were selected based on local CDC recommended criteria: relatively high HIV prevalence; density of high-risk groups; convenient location for HTC access by key populations; willingness to cooperate with CBOs; availability of HTC venues; and capacity for HIV rapid testing after training by local CDCs.

All participating CHCs established HIV rapid testing sites, displayed information, education and communication (IEC) materials for local HTC, and distributed information to general outpatients. Rapid testing was mainly provided in four ways: (1) doctors suggested outpatients with high-risk behaviors to have HTC, focusing on patients of dermatology, urology, obstetrics-gynecology and family planning departments; (2) doctors suggested inpatients, surgical patients and pregnant women to have HTC; (3) HTC were offered to clients actively seeking HTC; (4) HTC were provided for other clients who agreed to have HTC and were approached during CHC routine health work. For example, some FSWs were mobilized through CHC outreach intervention, and some clients through physical checks.

The HIV testing algorithm was based on national HTC guidelines [19], [20]. Inpatients who agreed to HTC were given pretest counseling and referred to the testing laboratory. Trained medical staff drew blood, typically using finger-stick techniques, and tested the samples using the Determine HIV-1/2® Rapid Test (Abbott Laboratories, USA). If the outcome was negative, the patient was told they were uninfected. If the test was reactive, the patient was told they were preliminarily positive. Post-test counseling was then given, focusing on the uncertainty of preliminary positive results and the role of confirmatory testing. At that time, venous blood samples were drawn and sent to the local CDC for repeated screening (mostly ELISA), HIV confirmation (Western blot), and syphilis tests. Medical staff at CHCs completed the HIV case information form during counseling. When CHCs received the confirmatory test results (usually took one to three weeks), medical staff met with the patient to explain the confirmatory test results, and also reported the case information to the web-based National AIDS Information System.

For outpatients, trained medical staff discussed risk factors with the patient to determine HTC eligibility. Individuals were suggested to have HIV testing if they had a history of high-risk sexual behavior, STIs, remunerated blood donation, receiving blood transfusion/products, drug use, or sexual partners with high-risk behaviors or PLHA. Tuberculosis (TB) patients with suspected clinical signs of HIV and children of PLHA were also tested. Information on offering free HIV testing in CHCs was also provided to patients reporting no risk behaviors for HIV. If these patients required testing, HTC was offered. If HTC was conducted, it followed the same procedure as for inpatients.

### Data collection in CHCs

Data was gathered from two sources. The first was aggregate administrative data on the number of patients who: were screened, received a positive screening, received a confirmatory test, were confirmed positive, were reported as a new HIV-positive case (excluding formerly reported cases), and received test results. Second, individual data was collected on each person tested, including general socio-demographic characteristics, reasons for the doctor visit, risk behaviors for HIV infection, and HTC results.

### Data collection in general hospitals

The China-Gates program also supported 15 program areas to improve the linkage between HIV screening performed in hospitals and confirmatory testing performed at local CDCs, in order to improve the HIV case detection efficiency in general hospitals. This was a part of the routine program activities that covered all general hospitals in the program areas. General hospitals reported to city CDCs on the number of patients who: were screened, received a positive screening, received a confirmatory test, were confirmed positive, were reported as a new HIV-positive case (excluding formerly reported cases), and received test results. We collected aggregate data from city CDCs at the city level.

### Data Analysis

We compared aggregate data from HTC in CHCs with that of HTC in general hospitals supported by the China-Gates program in the same pilot cities. Comparison focused on differences in proportions of screened positive, proportions of those receiving confirmatory test, efficiency of identifying new infections, and percentage of newly reported cases being followed up. Efficiency of identifying new infections referred to the ability to detect new HIV-infected persons as confirmed by WB; this was defined as the number of newly identified HIV infections divided by the number of HIV tests. Follow-up referred to the newly identified infections who were able to be reached by local CDCs and interviewed for a follow-up questionnaire after being reported through the national AIDS information system. This was defined as the number of confirmed HIV positive who were able to be followed up, divided by the number of reported screened HIV positives excluding those confirmed negatives.

Chi-square tests were used for comparisons in key indicators between the two categories of CHCs and general hospitals. Cumulative numbers of people living with HIV/AIDS and the population size of permanent residents for each pilot city at the end of 2012 were provided in Table 1 in order to help apprehend the regional variations of the HIV epidemic and variations of percentage of screened positives.

**Table 1:** HTC Cascade, Testing in CHCs and in General Hospitals in 2012.

Indicators	Location	City 1 N(%)	City 2 N(%)	City 3 N(%)	City 4 N(%)	City 5 N(%)	City 6 N(%)	City 7 N(%)	City 8 N(%)		Total			
										N(%)	Chi-square		p	
Participating CHCs (N)	-	5	4	5	4	4	11	4	5	41		-		-
Estimated outpatients seen per day	CHC	351	1811	164	57	305	351	72	191	419^1^		-		-
HIV screening tests (% of estimated patients)	CHC	7701(1.20%)	855(0.03%)	4184(1.75%)	904(4.35%)	1167(0.26%)	3330(0.24%)	4221(4.02%)	1247(0.45%)	23609(0.37%)		-		-
	General hospitals	2956767	1732796	854531	385358	523781	1144965	624276	1422470	9644944		-		-
Screened positive (% of screened)	CHCs	16(0.21%)	3(0.35%)	41(0.98%)	0(0%)	0(0%)	16(0.48%)	12(0.28%)	8(0.64%)	96(0.41%)		153.194		<0.001
	General hospitals	3515(0.12%)	1466 (0.08%)	2390 (0.28%)	237(0.06%)	357(0.07%)	530 (0.05%)	1221 (0.20%)	2154 (0.15%)	11870 (0.12%)				
Informed of positive screening results (% of screening positive)	CHC	16 (100%)	3(100%)	41(100%)	-(N/A)	-(N/A)	16(100%)	12(100%)	8(100%)	96(100%)		7.635^2^		0.006
	General hospitals	2694(76.6%)	1466(100%)	2390(100%)	237(100%)	355(99.4%)	506(95.5%)	1221(100%)	2126(98.7%)	10995(91.3%)				
Received confirmatory test (% of screening positive)	CHC	16(100%)	2(66.7%)	39(95.1%)	-(N/A)	-(N/A)	7(43.8%)	9(75.0%)	3(37.5%)	76(79.2%)		17.472		<0.001
	General hospitals	3066(87.2%)	1426(97.3%)	2071(86.7%)	237(100%)	355(100%)	527(99.4%)	794(65.0%)	1563(72.6%)	10039(84.6%)				
Confirmed positive (% of those confirmed – Positive predictive value)	CHC	10 (62.5%)	2(100%)	39(100%)	-(N/A)	-(N/A)	2(28.6%)	0(0%)	3(100%)	56(73.7%)		1.922		0.166
	General hospitals	2172(70.8%)	1088(76.3%)	1922(92.8%)	172(72.6%)	354(99.7%)	484(91.8%)	550(69.3%)	1296(82.9%)	8038(80.1%)				
Newly reported infections (% of confirmed positives)	CHC	9(90.0%)	2(100%)	39(100%)	-(N/A)	-(N/A)	1(50.0%)	0(N/A)	2(66.7%)	53(94.6%)		16.362		<0.001
	General hospitals	1106(50.9%)	941(86.5%)	1796(93.4%)	145(84.3%)	264(74.6%)	401(82.9%)	484(88.0%)	1002(77.3%)	6139(71.3%)				
Efficiency of identifying new infections	CHC	9(0.12%)	2(0.01%)	39(0.93%)	-(N/A)	-(N/A)	1(0.03%)	0(0%)	2(0.16%)	53(0.22%)		95.196		<0.001
	General hospitals	1106(0.04%)	941(0.05%)	1796(0.21%)	145(0.04%)	264(0.05%)	401(0.04%)	484(0.08%)	1002(0.07%)	6139(0.06%)				
Newly reported cases followed up (% of newly reported cases)	CHC	9(100%)	2(100%)	39(100%)	-(N/A)	-(N/A)	1(100%)	-(N/A)	2(100%)	53(100%)		0.585		0.444
	General hospitals	1103(99.9%)	897(97.3%)	1796(100%)	145(100%)	264(100%)	400(96.2%)	484(100%)	983(98.2%)	6072(98.9%)				
HIV Infection rate (No. of cumulatively reported PLWHA/city population size)	-	0.35‰	0.27‰	0.45‰	0.13‰	0.16‰	0.20‰	0.17‰	0.33‰	0.36‰		-		-

1 The mean value of number of estimated outpatients seen per day. ^2^ Fisher's exact test.

Individual data on each person tested was also analyzed to present frequency distributions in key democratic characteristics. Rate of screened positives was analyzed by age, gender, department recruited and risk behaviors. Chi-square tests were used to test the significance of differences.

### Ethics statement

This work was supported by the China-Gates Foundation HIV Cooperation Program (Grant number Opp49277). The Institutional Review Board of the National Center for AIDS/STD Control and Prevention, China CDC, approved the project and consent procedures. This project intended to explore the feasibility of increasing access of HTC by providing HTC in CHCs. All HIV consent and testing procedures followed relevant Chinese national guidelines. Patients discussed procedures with medical staff, and provided verbal consent prior to receiving HTC, which was documented in the patients' charts. Verbal consent was conducted in order to improve HIV testing rates. Medical staff in CHCs were specifically trained on confidentiality protections.

## Results

### HTC in CHCs

There were 23,609 patients who underwent HTC at the 42 CHCs. HTC performed by pilot CHCs accounted for 0.37% of all estimated clinic visits (0.03%–4.35% by site, Table 1). Overall positive screening prevalence was 0.41% (95% confidence interval [CI] 0.33%–0.49%, range 0.00%–0.98%), which was higher than general hospitals (0.17%). In two cities no PLHA were detected by CHCs.

The identification efficiency, or ability to detect new HIV-infected persons, was 0.22% (95% confidence interval [CI]: 0.16%–0.27%) in the pilot CHCs, 3.5 times higher than the 0.06% identification efficiency in general hospitals (Chi square test = 95.196, p<0.001). Across cities this ranged from 0.00% to 0.98% in CHCs and from 0.04% to 0.21% in general hospitals.

**Figure 1:**
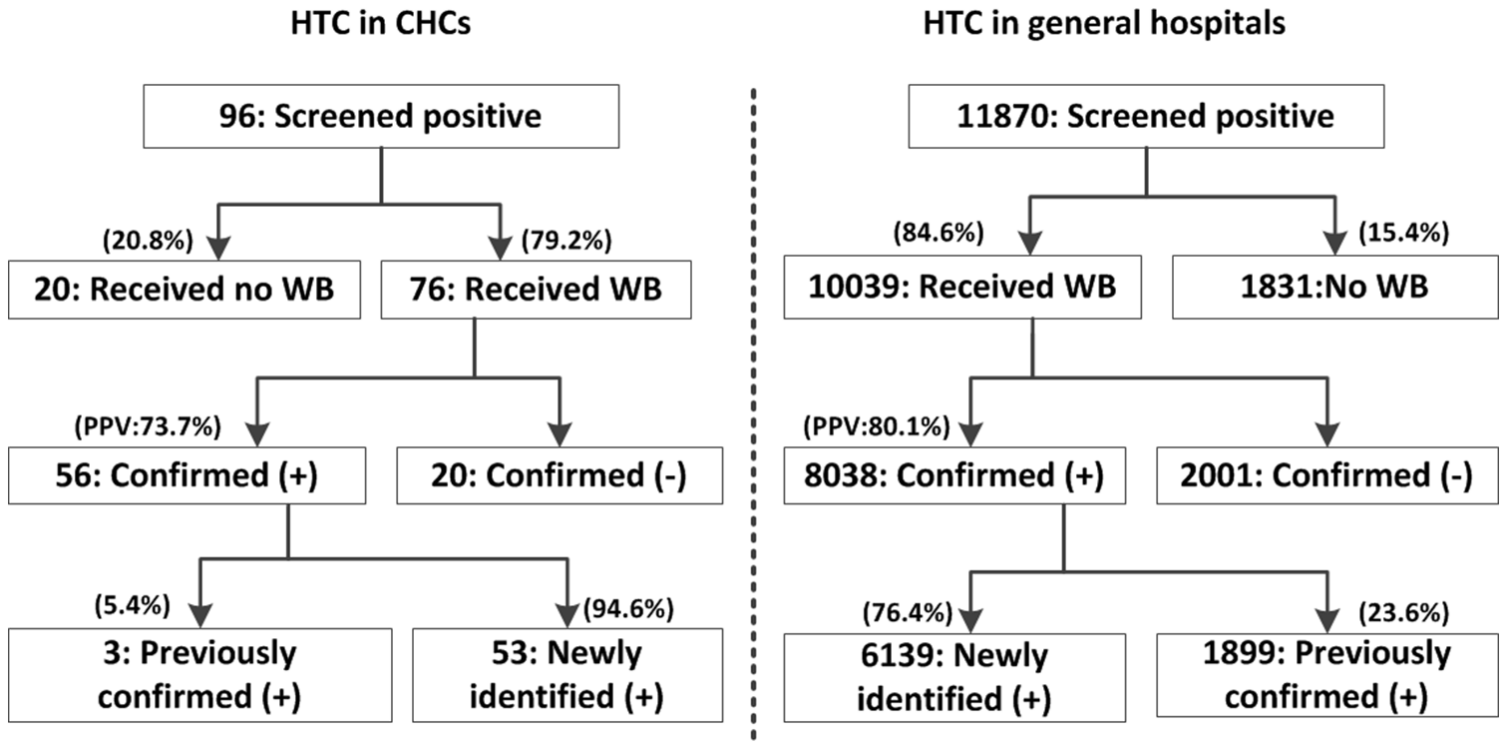
Flow chart of HTC cascade, testing in CHC's and in general hospitals in 2012.

As shown in Figure 1, attrition from screening to follow up was observed. Among patients who screened positive, 20.8% in CHCs did not received confirmatory test, higher than the 15.4% found in general hospitals (Chi-square test = 17.472, p<0.001); the positive predictive value for HTC in CHCs was 73.7% (95% confidence interval [CI] 63.8%–83.6%), which showed no significant difference from 80.1% in general hospitals (Chi-square test = 1.922, p = 0.166). The overall follow-up rate (the number of confirmed HIV positives who were followed up with, divided by the number of reported screened HIV positives excluding those confirmed negatives and those previously confirmed) for HTC was 72.6% (53/73) in CHCs, lower than 76.2% (6072/7970) in general hospitals (Chi-square test = 13.452, p<0.001).

### Patient Characteristics

There were 23,609 patients who were tested for HIV. However, only 87.5% (20,656) provided completed individual-level information.

Nearly two-thirds of those tested were between 20 and 40 years old; 72% were female (Table 2). Composition of clients mobilized for testing was consistent with the usage of basic public health and medical services in CHCs, with most coming from physical examinations (31.6%); urology, gynecology, perianal, and STI departments (27.9%); or outreach activities for high risk groups (17.5%).

**Table 2:** Characteristics of Clients Receiving HTC in CHCs, Jan-Dec, 2012.

Characteristics		Total		Screened HIV positive		Rate of screened positive
		n	%	n	%	%
**Age**	≦20	1978	9.6	6	7.3	0.30
	21–30	9058	43.9	20	24.4	0.22
	31–40	4138	20.0	18	22.0	0.43
	41–50	2578	12.5	15	18.3	0.58
	≧51	2884	13.9	23	28.0	0.80
**Gender**	Male	5896	28	54	65.9	0.92
	Female	14760	72	28	34.1	0.19
**Department**	Physical examination	6532	31.6	20	24.4	0.31
	Outreach intervention	3623	17.5	15	18.3	0.41
	VCT	1260	6.1	7	8.5	0.56
	Surgical patients	1269	6.1	0	0.0	0.00
	Pregnant women	2199	10.6	1	1.2	0.05
	Outpatients in key departments^1^	5769	27.9	39	47.6	0.68
**Risk behaviors** 2	STD related symptoms	4021	19.5	35	-	0.87
	MSM	177	0.9	16	-	9.04
	Injecting drug use	104	0.5	10	-	9.62
	Commercial sex	4149	20.1	22	-	0.53
	Multiple or concurrent sexual partners	6639	32.1	34	-	0.51
	Received blood transfusion/blood products	1675	8.1	3	-	0.18
	History of paid blood donation	239	1.2	0	-	0.00
	No risk behaviors reported	6913	33.4	8	-	0.12

1 Key departments were primarily dermatology, urology, obstetrics-gynecology and family planning departments. There was variation across CHCs due to different organizational structures.

2 Totals may add to more than 100% due to ability to choose multiple answers.

The HIV positivity rate was higher among males and older people than among females (Chi-square test = 56.187, p<0.001) and younger people (Chi-square test = 21.609, p<0.001). The rate was also higher among patients from urology, gynecology, STI or perianal, fever or trauma departments in CHCs (0.68%) than among high risk groups participating in VCT (0.56%) and outreach activities (0.41%), pregnant women (0.05%)and surgical patients (0.00%)(Table 2).

## Discussion

This pilot project shows that integrating HIV rapid testing with CHC routine services is feasible, results in a relatively higher detection rate than general hospitals, and can provide good referral quality. Offering HIV rapid testing in CHCs significantly increased HTC usage among attendees with risk behaviors for HIV who typically use basic CHC public health and medical services. This suggests CHCs successfully integrated HTC into the provision of routine public health services. While HTC testing as a percentage of CHC visits was low (<5%), the pilot was only for one year. As connections are built between patients and testing services, the percentage of visits that include HTC may increase [9], [13].

Concerns have been voiced in China that CHC-based HTC will not be cost-effective [21], as HIV prevalence among attendees in health care facilities are considered to be low [22]. Our results indicate that CHCs can effectively identify new HIV infections, with case-finding rates 3.5 times higher than general hospitals. This may be due to CHC's targeted testing of those with high-risk behaviors, in contrast to the fact that general hospitals test all inpatients, surgical patients, and pregnant women, regardless of risk behaviors. In addition, variation in HIV infection rates was observed across groups of attendees from different departments who were tested. We found outpatients in key departments had higher HIV rates than individuals in VCT clinics or key populations mobilized through outreach. Typically, higher HIV rates would be expected from VCT clinics or outreach intervention [22], due to the higher risk behavior of the populations involved. This indicates the value of utilizing HIV testing mobilization among outpatients of CHCs as a means of case finding.

In addition to successful case finding, pilot CHCs effectively linked newly discovered PLHA to care. In fact, follow-up rates for newly discovered infections were high in CHCs with follow up on all newly diagnosed infections. However, the overall follow-up rate for screened positives was lower than at general hospitals. It was also significantly lower than the linkage-to-care rate indicated in a recent meta-analysis of community-based testing approaches [23]. In this meta-analysis, 17 studies presented an overall rate of 80% (95% CI: 75%–85%) receiving CD4 testing after diagnosis. However, this meta-analysis does not clarify the definition of HIV diagnosis or whether diagnosis needs to be confirmed with WB test. In our study, the low follow-up rate is mainly due to a high proportion of screened positives not receiving confirmatory test, as reflected by the results that over 20% of individuals who screened positive at CHCs did not receive a confirmatory WB test. This may be due to a high proportion of testing individuals who had previously been confirmed positive were not offered WB test. This can be reflected in that 23.6% of confirmed positives in general hospitals had been confirmed previously, compared to only 5.4% in CHCs. Additionally, blood samples of some screened positives in CHCs were repeatedly tested in CDCs using ELISA. If ELISA was negative, WB testing was not offered. Due to limitations of the data collected, we could not calculate the exact number of such cases. Furthermore, some loss to follow-up in CHCs could have been due to the mobility of the population or because participants gave fake or incomplete contact information [24]. General hospitals typically conduct HTC with inpatients, who are easier to follow-up with than the outpatients that CHCs mobilized for HTC. Improved training for medical staff could mitigate some of these issues.

The positive predictive value for HTC in CHCs is around 74%. Compared with the PPV obtained in some international field studies that ranged from 40% to 100% due to diverse contexts of HIV prevalence [25], [26], [27], [28], [29], the PPV of our study tends to be consistent. In addition, the PPV varied across pilot areas from 0% to 100%. As all sites received the same test kits, differences of PPV may be associated with the testing quality. Most CHCs were providing rapid testing for the first time [30]. However, even in general hospitals, only 80% of those who received a confirmatory test were confirmed positive, with variation across cities from 70% to 100%. This suggests variation across CHCs was not wholly quality-related, since studies have demonstrated a relatively high false positive rate in low prevalence populations with rapid tests [30], [31]. As such, it remains important to communicate to patients the uncertainty of preliminary positive results and the necessity of confirmatory testing. In resource constrained settings, the World Health Organization (WHO) recommends using a two or three HIV rapid test algorithm instead of traditional ELISA plus confirmatory test after a system of evaluation and continuous quality assurance [32]. However, the current HIV testing and diagnosis guidelines in China still require confirmatory test for HIV diagnosis – usually a WB test – unless the HIV prevalence is higher than 5% in which case two screening test algorithms (Rapid test or ELISA) can be applied [19].

This study is the first conducted in China to explore the feasibility of HTC in CHCs with a large sample size. However, our findings should be interpreted within project limitations. First, absence of patient IDs prevents linking data across individual and aggregate variables and from conducting an analysis of exact case detection efficiency by subgroup. Second, data was not collected on the proportion of CHC patients who had high-risk behavior and who accepted HTC.

China has a solid national policy foundation for including CHCs in the HIV/AIDS response. However, challenges remain. Some CHCs are funded completely by the government, with performance-based staff salaries. As HTC does not influence salary, many providers perceive it as extra work and are reluctant to conduct HIV/AIDS services [18]. Furthermore, local health administrative departments hold varying attitudes towards conducting HTC at CHCs. They have concerns about the willingness of communities to participate, cost-effectiveness of CHC case-finding, and protection of PLHA's private medical information. Patients may worry about stigma, discrimination, exposure, and the technical capacity of CHC staff [14], [18].

This project demonstrates that CHCs can integrate HTC into routine work, expand HTC access, and increase HIV case finding. Half of the PLHA in China are unaware of their HIV status [1], which remains a major challenge for national HIV prevention efforts [33]. A key solution is expanding access to HTC [34]. In conjunction with China's ongoing medical health system reform, we expect that the HTC responsibilities of CHCs will become more clear and specific. Eventually the focus may move from testing patients with risk factors to testing all patients. With additional training and support, CHCs could play a significant role in expanding access to HTC and promoting HIV case finding in China.
